# Primary nasopharyngeal Hodgkin's disease: case report and literature review

**DOI:** 10.1186/1752-1947-4-116

**Published:** 2010-04-26

**Authors:** Youssef Bensouda, Kawtar El Hassani, Nabil Ismaili, Issam Lalya, Saber Boutayeb, Nourredine Benjaafar, Brahim Khalil El Gueddari, Hassan Errihani

**Affiliations:** 1 Department of Medical Oncology, National Institute of Oncology, Rabat, Morocco; 2 Department of Radiotherapy Oncology, National Institute of Oncology, Rabat, Morocco

## Abstract

**Introduction:**

Primary Hodgkin's disease of the nasopharynx is a rare and uncommon event. It has a relatively favorable prognosis and represents less than 1% of all documented cases of Hodgkin's disease.

**Case presentation:**

A 40-year-old Arabic man presented initially with bilateral nasal obstruction, which was then followed by a significant involvement of his bilateral cervical lymph nodes. His nasopharyngeal biopsy together with immunohistochemistry analysis showed negative expressions of CD15, CD20 and CD3, but positive expressions of CD30 and epithelial membrane antigen. This confirmed the diagnosis of nasopharyngeal Hodgkin's disease of a mixed cellularity subtype. The disease was at stage IIEA. Our patient received four cycles of chemotherapy, which yielded a 75% response. This was followed by irradiation of his Waldeyer's ring and supraclavicular lymph nodes. He remains in good local control after 30 months of follow-up.

**Conclusion:**

The literature review and our case report discuss the optimal management of this rare and atypical localization of Hodgkin's disease, which should be differentiated from lymphoproliferations associated with Epstein-Barr virus and non-Hodgkin's lymphoma.

## Introduction

The lymphoid tissues of Waldeyer's ring, including the nasopharynx, are rarely involved in Hodgkin's disease (HD). Primary nasopharyngeal presentation is extremely rare, occurring in less than 1% of all reported cases of HD. With appropriate treatment the prognosis for this particular type of HD is favorable. Most documented cases of HD are either stage I or II.

We present the case of a patient with primary nasopharyngeal HD which was managed by a combination of sequential chemotherapy and radiotherapy. This treatment yielded optimal local control after 30 months of follow-up.

We reviewed the literature and considered questions about the rarity of this case, the optimal management of its atypical localization, and the need for immunohistochemistry (IHC) analysis in differentiating HD from non-Hodgkin's lymphoma (NHL) and lymphoproliferations associated with Epstein-Barr virus (EBV).

## Case presentation

In October 2005, a 40-year-old Arabic man with a long history of smoking presented at the National Institute of Oncology for a consultation. Over a period of one year, he had developed progressive bilateral nasal obstruction with a secondary cervical left mass that was associated with headaches and hypoacusis. No fever, pruritus, sweat or weight loss was noted. His full blood count, biochemical tests and erythrocyte sedimentation rate were all normal. A clinical examination of our patient found his cervical left lymph nodes measured 8 × 5 cm and right submaxillary lymph nodes measured 5 × 4 cm.

**Figure 1:**
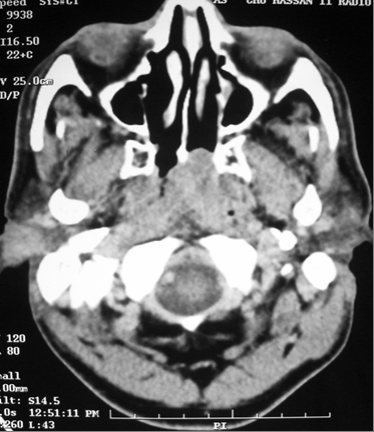
**Computed tomography scan before treatment**. Left nasopharyngeal mass involving the parapharyngeal space but without extracranial or bone involvement.

**Figure 2:**
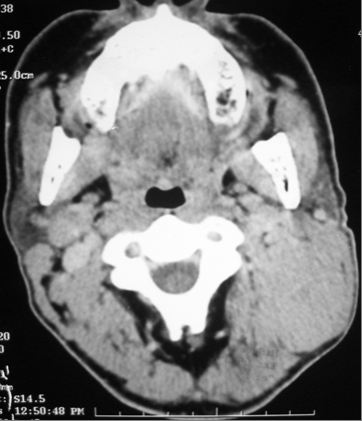
**Computed tomography scan before treatment**. Left nasopharyngeal mass involving the parapharyngeal space but without extracranial or bone involvement.

A computed tomography (CT) scan of his nasopharynx and nasofibroscopy revealed a posterolateral nasopharyngeal mass and bilateral cervical lymph nodes (Figures 1 and 2). A nasopharyngeal biopsy and IHC analysis confirmed the diagnosis of HD of a mixed cellularity type (CD30+, epithelial membrane antigen positive [EMA+], CD15-, CD20-, CD3- and cytokeratin).

The results of his bone marrow biopsy, and chest, abdomen and pelvic CT scans were all normal. His disease was staged at IIEA according to the Ann Arbor classification system.

**Figure 3:**
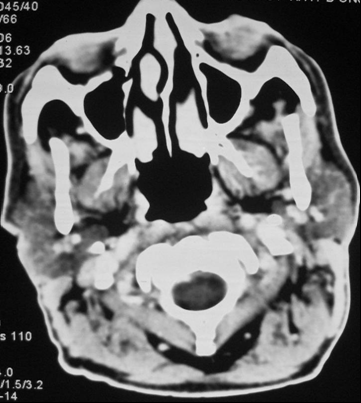
**Computed tomography scan after chemotherapy**. Complete regression of nasopharyngeal mass with cervical necrosis lymph nodes.

Our patient was then treated using four cycles of chemotherapy every three weeks with an alternating regimen of cyclophosphamide, Oncovin (vincristine), prednisolone (COP) and Adriamycin (doxorubicin), bleomycin, vinblastine (ABV) (day one: COP; day eight: ABV). His radiological evaluation after the fourth cycle showed a complete resolution of the nasopharyngeal mass and a 75% response in his cervical nodes (Figure 3). The treatment was then completed by irradiation of his Waldeyer's ring and cervical lymph nodes with a total dosage of 36 Gy. Our patient remains in good local control after 30 months of follow-up.

## Discussion

We report the rare case of a patient with primary nasopharyngeal HD which was successfully managed by a sequential combination of chemotherapy and radiotherapy.

Hodgkin's disease localized in the head and neck regions is mostly seen in the nodal tissues. Extranodal involvement is rarely reported. According to Eavey and Goodman, only two cases of nasopharyngeal HD have been identified in a study of 500 cases of HD localized in the head and neck regions [1].

Primary nasopharyngeal presentation is exceptional in HD cases, as it occurs in less than 1% of all reported HD localizations. In another case report, Anselmo *et al. *reported only seven cases (0.32%) of nasopharyngeal HD in a large retrospective study involving 2150 cases of HD documented over a period of 24 years [2].

Compared with HD, NHLs are more frequently localized in the head and neck regions. A retrospective review of 311 head and neck lymphomas reported only three cases (4%) of HD whereas NHL cases were 24% of the total [3]. Fewer than 90 cases of HD with nasopharyngeal involvement have been reported in the literature worldwide; but only 20 of these cases primarily involved the nasopharyngeal region [2,4-12] (Table 1).

**Table 1:** Case reports of primary nasopharyngeal Hodgkin's disease in the literature: patient characteristics.

Author	Age	Sex	Stage	Treatment	Response	DFS
Our Case	40	M	IIE Aa	CT 4 × COP/ABV→RT	PR 75%	30 m
Atasoy 2006 [4]	45	F	IE A	RT	CR	26 m
Kochbati 2006 [5]						
Case 1	41	M	IE Aa	RT	CR	3 y
Case 2	36	M	IIE Aa	CT 3 × ABVD→RT	PR 90%	6 m
Case 3	77	F	IIE Aa	CT 3 × ABVD→RT	PR	nv
Hollander 2004 [6]	36	M	IIE	CT 6 × ABVD→RT	CR	6 m
Takashima 2003 [7]	46	M	IE A	CT 3 × ABVD→RT	CR	6 m
Anselmo 2002 [2]						
Case 1	44	M	IIE A	RT (mantle)	CR	24 y
Case 2	47	M	IIE A	CT 2 × ABVD→RT	CR	6 y
Case 3	40	M	IIE A	CT 4 × ABVD→RT	CR	8 y
Case 4	34	M	IE A		CR	6 y
Case 5	25	M	IIE A	CT 2 × ABVD→RT	CR	6 y
Case 6	43	M	IIE A	CT 4 × ABVD→RT	CR	3 m
Case 7	29	M	IIE A	CT 2 × ABVD→RT	CR	1 m
Herrmann 2002 [8]	43	F	IE A	Compl resection	CR	14 m
Folz 2000 [9]	45	M	IIE A	Partial resection	PR	nv
Molony 1998 [10]	46	M	IIE Aa	CT 3 × CVPP→RT	CR	nv
Daniel 1988 [11]	23	M	IE	Adenoidectomy + RT	CR	7 y
O'Reilly 1987 [12]	62	M	IE A	RT	CR	14 m

DFS: disease-free survival, M: male, F: female, CT: chemotherapy, RT: radiotherapy, ABVD: Adriamycin (doxorubicin), bleomycin, vinblastine, dacarbazine, COP: cyclophosphamide, Oncovin (vincristine), prednisolone, Compl: complete, CR: complete response, PR: partial response, m: months, y: years, nv: not valuable.

Hodgkin's disease is predominantly seen in male patients, and mixed cellularity is its most frequent histological subtype [2,12]. Because EBV was found in the majority of cases reported, nasopharyngeal HD should be differentiated from lymphoproliferations associated with EBV.

Before IHC analyses became widely available, some authors speculated that nasopharyngeal HD localization might have been under-diagnosed [1,12]; but judging from its continued rarity, it appears that this claim is false.

To confirm a diagnosis of nasopaharyngeal HD, an IHC analysis is necessary. As found in the majority of cases, Reed-Stenberg cells with positive expressions of CD30 and/or CD15, and negative expressions of CD20, CD3 and CD4, corroborate the diagnosis (Table 2).

**Table 2:** Case reports of primary nasopharyngeal Hodgkin's disease in the literature: subtype and immunohistochemistry pattern.

Author	Subtype HD	CD15	CD30	EMA	LMP1	CD3	CD20	CD45
Our case	MC	-	+	+		-	-	
Atasoy 2006 [4]	MC	+	+	-	-	-	-	
Kochbati 2006 [5]								
Case 1	MC	-	+		+	-	-	
Case 2	MC	+						
Case 3	MC	+	+	-	-		+	
Hollander 2004 [6]	MC	+	+				+	
Takashima 2003 [7]	Classical							
Anselmo 2002 [2]								
Case 1	MC							
Case 2	MC							
Case 3	LP							
Case 4	LP		+			+	-	-
Case 5	Interfollicular							
Case 6	NS	+/-	+					
Case 7	LP	+	+					
Herrmann 2002 [8]	NS	+	+					
Folz 2000 [9]	LP	+	+					
Molony 1998 [10]	Classical	+	+	+/-			-	-
Daniel 1988 [11]	MC							
O'Reilly 1987 [12]	LP							

EMA: epithelial membrane antigen, MC: mixed cellularity, NS: nodular sclerosis, LP: lymphocyte predominance.

The treatment of nasopharyngeal HD should be similar to that used in other HD localizations, which involve a sequential combination of chemotherapy and radiotherapy. We believe that four cycles of a chemotherapeutic regimen (Adriamycin [doxorubicin], bleomycin, vinblastine, dacarbazine [ABVD] polychemotherapy) is the appropriate standard regimen; and involved field radiotherapy with target volume given as an intermediate dosage (25 to 40 Gy) targeting the Waldeyer's ring and cervical lymph nodes should be the first line of treatment for patients with nasopharyngeal HD. Furthermore, exclusive irradiation should be proposed for isolated cases of nasopharyngeal HD that do not involve the cervical lymph nodes and do not present with general symptoms.

## Conclusion

From data gathered through our case and literature review, we conclude that nasopharyngeal HD is an atypical and rare localization. The majority of cases reported are localized at stage I or II; our patient was diagnosed at stage IIEA.

The optimal management of primary nasopharyngeal HD is still unclear. Its treatment, first with chemotherapy followed by involved field irradiation, appears to be an adapted therapy, especially when cervical lymph nodes are involved. This combined treatment is associated with the long-term cessation of the disease.

## Consent

Written informed consent was obtained from the patient for publication of this case report and any accompanying images. A copy of the written consent is available for review by the Editor-in-Chief of this journal.

## Competing interests

The authors declare that they have no competing interests.

## Authors' contributions

YB was the principal physician who managed our patient, performed the literature research, and wrote the manuscript. KEH helped write the manuscript and performed the literature review. NI helped write the manuscript and analyzed the final results of our patient's examinations. IL helped with modifications and revisions to the manuscript, also in the final conception of the article, principally in the redaction of the manuscript. SB managed our patient's chemotherapy cycles and analyzed the literature. NB performed and approved the radiotherapy part of our patient's treatment. BKEG analyzed and interpreted our patient's data for the radiotherapy section of the manuscript. HE approved the treatment and analyzed the literature data. All authors read and approved the final manuscript.
